# Comparison of Primary Care Experiences and Outpatient Health Service Utilization Among Black and Latino Homeless-Experienced Veterans: An Analysis of Patient-Centered Medical Homes

**DOI:** 10.1177/21501319251382520

**Published:** 2025-11-09

**Authors:** Melissa Chinchilla, Audrey L. Jones, Aerin DeRussy, Michael F. Green, Lillian Gelberg, Alexander S. Young, Jack Tsai, Sonya E. Gabrielian, Stefan G. Kertesz

**Affiliations:** 1 University of California Los Angeles, USA; 2 Veterans Affairs Greater Los Angeles Healthcare System, CA, USA; 3 University of Utah School of Medicine, Salt Lake City, USA; 4 Birmingham VA Medical Center, AL, USA; 5 Greater Los Angeles Healthcare System, CA, USA; 6 VA Desert Pacific Mental Illness Research, Education, and Clinical Center, Los Angeles, CA, USA; 7 U.S. Department of Veterans Affairs, Washington, DC, USA; 8 University of Texas Health Science Center at Houston, USA; 9 Yale University School of Medicine, New Haven, CT, USA; 10 University of Alabama at Birmingham School of Medicine, USA; 11 University of Alabama at Birmingham School of Public Health, USA

**Keywords:** healthcare, homelessness, needs and experiences of care, primary care, veterans

## Abstract

**Objectives:**

To examine whether, among persons with recent homeless experience, minoritized clients rate primary care differently from non-minoritized clients in Veterans Affairs (VA) mainstream and homeless tailored clinics, through the largest survey of homeless-experienced Black, White, and Latino Veterans to date.

**Methods:**

Surveys were collected from HEVs in Homeless-tailored Patient-Aligned Care Teams (HPACTs) and mainstream Patient Aligned Care Teams (PACTs; n = 4894). We tested multivariable associations between race/ethnicity, clinic (HPACT vs mainstream-PACT), and their interaction, on care experience ratings and service utilization.

**Results:**

There were no major differences in care ratings by race/ethnicity; medical and social vulnerability factors were associated with worse ratings. Black HEVs rated team Cooperation and Access/Coordination modestly better compared to White HEVs, while being Latino was nonsignificant. HPACTs were rated higher than mainstream-PACTs. Better Access/Coordination ratings were associated with more primary care (+1.12 additional visits per point increase) and mental health outpatient visits (+4.37 additional visits per point increase).

**Conclusions:**

In VA primary care, homeless-tailored clinics outperformed mainstream ones while racial/ethnic differences in ratings were minor. Optimizing perceived Access/Coordination of services may offer a path to increased service use.

## Introduction

Black and Latino individuals experience elevated rates of homelessness, health disparities, and barriers to care when compared to non-Latino Whites.^1-4^ Among U.S. Veterans, Black and Latino persons represent 13% and 9% of the population but are overrepresented among homeless-experienced Veterans (HEVs; 31.3% and 13.2%, respectively).^5^ Compared to non-homeless Veterans, HEVs report poorer experiences with primary care^6^ and higher rates of Emergency Department (ED) use.^7^ Department of Veteran Affairs (VA) developed a primary care model tailored to address the medical needs of HEVs.^8-10^ However, it is unknown how well it serves Black and Latino HEVs.^11^

VA reorganized primary care services into Patient Aligned Care Teams (PACTs). PACTs are team-based patient-centered medical homes that provide whole person care.^12^ In 2012, VA modified the PACT model to serve HEVs,^10^ coined “Homeless Patient Aligned Care Teams (HPACTs).”^13^ HPACTs enroll HEVs with experiences of homelessness and provide services to address access barriers and social determinants of health (SDoH), including housing.^10,14^ HPACTs increase care access through flexible scheduling (ie, extended hours and walk-in appointments); alignment between mental health and social services; onsite supports (eg, food pantries); and smaller panel sizes that enable providers to dedicate greater time and resources to patients.^13^ HEVs enrolled in HPACTs are more likely than those in PACTs to report favorable primary care experiences.^6,14,15^ HPACT empanelment is also associated with increases in primary care use and reductions in ED visits and hospitalizations.^16^ However, whether such positive experiences equitably extend to minoritized HEVs remains unknown. To inform this gap, our study uses national survey data to compare demographic and clinical differences, primary care experiences, and outpatient health service utilization among a sample of Black, Latino, and White HEVs who received care in HPACT and mainstream-PACT programs.^17^

## Methods

We used data collected through the “Primary Care Quality and Homeless Service Tailoring (PCQ-HoST)” study,^17,18^ a national survey of HEVs’ health needs and experiences in HPACT versus mainstream-PACT. PCQ-HoST was implemented in 2017 at 26 VA Medical Centers across 20 states. This data was linked to VA’s Electronic Health Record (EHR) to identify participants’ health service utilization. Our study was approved by VA’s Central Institutional Review Board.

### Participants

Veterans were eligible for recruitment if they had evidence of homelessness as identified by International Classification of Diseases (ICD) codes,^19^ attended ≥2 primary care visits in 24-months at the same site, and were coded as empaneled by a single HPACT or mainstream-PACT. Surveys were mailed to 14 340 HEVs. Respondents (n = 5766) included 3394 HEVs from HPACTs (59%) and 2372 from mainstream-PACTs (41%).^15^ For our study, non-Latino individuals whose race was missing or reported as other (n = 777) and participants whose gender was listed as missing or other (n = 24) were excluded due to small sample sizes. Our final sample (n = 4894) included 3 groups, Latino/Hispanic (n = 595), henceforth Latino (inclusive of all races), non-Latino Black (2181), and non-Latino White (2118).

### Covariates

The PCQ-HoST study included covariates derived from the Behavioral Model for Vulnerable Populations.^20^ The framework identifies factors that *predispose* individuals to access services (eg, demographics), which interact with *enabling* factors that allow or impede an individual’s access to healthcare services (eg, source of care) and *needs* for services (eg, perceived and evaluated health) that influence health service use *behaviors* and *outcomes.*^20^ Predisposing characteristics included age, sex, race, and ethnicity. Enabling characteristics included unsheltered homelessness (ie, ≥1 night in last 6-months spent in a place not meant for human habitation) and EHR-evidence of being assigned to a HPACT versus mainstream-PACT. Need characteristics included self-reported psychological distress, based on scoring ≥10 out of 24 on a combination of 4 depression/anxiety items from the Patient Health Questionaire-4 (PHQ-4) and 2-items assessing psychotic symptoms from the Colorado Mental Health Symptom Index (range = 0-24, Cronbach α = .84).^21^ Severe chronic pain was assessed with a single item from the Brief Chronic Pain Questionnaire focused on pain lasting ≥3-months with an average past-week pain severity rating of ≥7 on a 10-point pain scale.^22,23^ Substance use was captured by 2-Item Conjoint Screen (TICS),^24^ which queries past-year excess alcohol or drug use, and personal experience of an overdose in the past 3-years requiring medical attention.^17^

### Outcome Variables

Patient experience with primary care was captured by 4-scales (Supplemental Table 1)^25^: (i) Patient-clinician relationship (“Relationship,” 15-items); (ii) cooperation among clinicians (“Cooperation,” 3-items); (iii) accessibility and coordination of services (“Access/Coordination,” 11-items); and (iv) ability of clinic to meet homeless needs (“Homeless-specific Needs,” 4-items). Scales are scored using a Likert scale, “strongly agree = 4,” “agree = 3,” “disagree = 2,” “strongly disagree = 1,” and “I don’t know = 0.” Reverse-scoring is done for items where agreement indicates a worse experience.^15^

Health service utilization was captured for 24-months (12-months before and after survey date) using clinic stop codes. VA outpatient mental health and SUD visits captured behavioral health service use. Mental health visits were assessed for participants with EHR-evidence of a corresponding mental health diagnosis (ie, psychosis, depression, anxiety, or post-traumatic stress disorder) in the 24-months prior to survey completion, or whose survey recorded psychological distress. SUD visits were assessed for participants with active alcohol or drug use, or who had a SUD diagnosis (ie, alcohol or drug use disorder) in the EHR over the 24-months prior to survey completion. Primary care service utilization was examined across participants, regardless of self-reported or documented medical need.

### Study Design

Analyses were performed in 4 steps. First, we used analysis of variance (ANOVA) to compare characteristics of White, Black, and Latino HEVs enrolled in HPACTs versus mainstream-PACTs. Given that these analyses were intended to be exploratory and hypothesis-generating, statistical models did not control for multiple comparisons. Second, general linear models, controlling for covariates and site-level random effects, tested racial/ethnic differences in primary care experience and healthcare utilization by PACT type. An interaction term (race/ethnicity × PACT type) examined whether racial/ethnic differences in primary care experience varied by enrollment. Third, for significant interactions, models were stratified by race/ethnicity. Last, we re-ran the utilization models, controlling for patient experience, to assess its independent association with primary care, mental health, and SUD service utilization. All models included a random effect to account for clustering within 26 VA facilities. Significance was assessed at *P* < .05. Analyses were conducted using Stata 18.

## Results

### Sample Characteristics

HEVs’ predisposing, enabling, and need characteristics significantly (*P* < .05) differed by race/ethnicity and clinic type (Table 1).

**Table 1.** Characteristics of White, Black, and Latino Veterans with Homeless Experience in HPACT and mainstream-PACT Primary Care Settings.

Variable	HPACT White (n = 1249)	HPACT Black (n = 1282)	HPACT Latino (n = 354)	PACT White (n = 869)	PACT Black (n = 899)	PACT Latino (n = 241)
Predisposing						
Gender***						
Male	95.4% (1,191)	94.7% (1,214)	92.4% (327)	86.7% (753)	86.9% (781)	83.0% (200)
Female	4.6% (58)	5.3% (68)	7.63% (27)	13.4% (116)	13.1% (118)	17.0% (41)
Age***						
18-54	26.4% (330)	23.1% (296)	38.4% (136)	23.6% (205)	20.6% (185)	38.6% (93)
55-65	54.4% (679)	61.2% (785)	48.0% (170)	37.4% (325)	52.7% (474)	41.9% (101)
66+	19.2% (240)	15.7% (201)	13.6% (48)	39.0% (339)	26.7% (240)	19.5% (47)
Enabling						
Unsheltered nights (past 6 months)***						
0	85.5% (1,068)	83.0% (1,064)	82.8% (293)	90.1% (783)	87.2% (784)	83.8% (202)
1 or more	14.5% (181)	17.0% (218)	17.2% (61)	9.9% (86)	12.8% (115)	16.2% (39)
Need						
Psychological distress^a^***	28.7% (331)	31.8% (374)	42.59% (138)	31.0% (252)	28.5% (233)	41.07% (92)
Alcohol/drug use ***						
Alcohol	28.2% (348)	34.4% (429)	30.2% (105)	22.5% (194)	28.7% (252)	34.9% (83)
Drug	12.3% (152)	14.6% (128)	14.1% (49)	7.3% (63)	18.8% (235)	15.55% (37)
Both	8.2% (102)	13.2% (169)	9.9% (35)	4.3% (37)	10.1% (91)	12.9% (31)
Overdose	9.8% (121)	4.6% (58)	9.5% (33)	7.0% (60)	3.8% (34)	8.0% (19)
Severe chronic pain^b^***	28.5% (356)	42.5% (545)	37.3% (132)	34.5% (300)	41.1% (369)	46.9% (113)
Outcomes						
Patient experience ratings (scale 1-4)						
Relationship***	3.20 (0.58)	3.21 (0.56)	3.19 (0.57)	3.07 (0.62)	3.11 (0.56)	3.05 (0.65)
Cooperation***	2.84 (0.76)	2.80 (0.73)	2.72 (0.75)	2.61 (0.83)	2.72 (0.73)	2.64 (0.80)
Access/coordination***	3.08 (0.53)	3.08 (0.52)	3.06 (0.53)	2.94 (0.57)	2.98 (0.50)	2.92 (0.65)
Homeless-specific needs***	3.04 (0.62)	3.04 (0.59)	3.00 (0.64)	2.86 (0.67)	2.85 (0.65)	2.82 (0.78)
Health service utilization, past 2 years (# of visits)						
Primary care***	10.08 (10.69)	10.02 (9.10)	10.32 (8.86)	12.12 (12.99)	10.39 (10.25)	10.44 (9.93)
Mental health outpatient*	14.39 (27.69)	13.58 (30.22)	15.73 (28.18)	12.15 (26.85)	11.30 (25.70)	15.63 (30.62)
SUD outpatient*	5.99 (19.05)	6.39 (23.22)	6.31 (24.00)	3.74 (16.59)	4.42 (19.84)	4.22 (15.79)

a *Psychological distress*: Based on combining the 4-item PHQ-4 and 2 items related to psychotic symptoms from the Colorado Mental Health Index, with a score of >10 (sum range 0-24), counted as “Severe” when sum >10, see Methods Section.

b *Severe Chronic Pain*: Participants who reported having bodily pain that of more than 3 months duration coupled with current pain ≥7 on a 0-10 scale.

Outcome measures presented as means with standard deviations.

*** *P* < .001. **P < .01. *P < .05; *P*-values shown are for comparison across the 6 columns.

#### Predisposing

*Gender.* Across racial/ethnic groups, HPACTs served a lower proportion of female HEVs than PACTs. *Age.* Regardless of clinic type, Latino HEVs were younger than White or Black HEVs. In non-Latino groups, PACT patients were older (27%-39% over age 65 years) than HPACT patients (16%-19% over age 65 years).

#### Enabling

*Unsheltered Homelessness*. All racial/ethnic groups in HPACT were more likely to be unsheltered compared to their PACT counterparts. Black and Latino HPACT patients were more likely to report unsheltered homelessness in the past 6-months (17%), compared to White patients (15%). Latino patients had the highest rate (16%) of unsheltered homelessness in PACT (9%-13%).

#### Need

*Chronic Pain*. Black and Latino HEVs were more likely than White HEVs to report severe, chronic pain. In PACTs, 41% and 47% of Black and Latino patients, respectively, had severe chronic pain, compared to 35% of White patients. In HPACTs, 37% and 43% of Black and Latino patients, respectively, had severe, chronic pain, compared to 29% of White patients. *Mental Health.* Latino patients were more likely than Black and White patients to experience psychological distress. In PACTs, 41% of Latino patients had psychological distress, compared to 29% and 31% for Black and White patients. In HPACTs, 43% of Latinos experienced psychological distress, compared to 29% and 32% for White and Black patients. *Substance Use*. Across HPACT and PACTs, the prevalence of drug and/or alcohol use was higher among Black and Latino HEVs, compared to White HEVs. Across all groups, HPACT patients were more likely than PACT patients to report an overdose. However, regardless of clinic, Black HEVs were less likely than White or Latino HEVs to report a prior overdose. White HEVs in PACT were less likely to report drug use (7%) than White HEVs in HPACT (12%). In contrast, both Black and Latino HEVs in PACT had higher prevalence (19%-16%) of drug use compared to their HPACT counterparts (15%-14%). Both White and Black HEVs in PACT were less likely to report high rates of both alcohol and drug use (4%-10%) compared to their HPACT counterparts (8%-13%). In contrast, Latino HEVs in PACT (13%) were more likely to report both alcohol and drug use when compared to HPACT (10%).

##### Outcomes

Across patient experience ratings, mean unadjusted scores on all scales were higher among HPACT respondents compared to PACT (Table 1). There were minimal differences by race/ethnicity. Mean health service utilization for both SUD and mental health visits was slightly higher for HPACT participants. The opposite was evident for primary care.

### Racial/Ethnic Differences in Patient Experience

After adjustment for covariates, race/ethnicity was significant (*P* < .05) for 2 primary care experience measures: Cooperation and Access/Coordination (Table 2).

**Table 2.** Patient Experience Ratings Using the 2018 Primary Care Quality and Homeless Service Tailoring (PCQ-HoST) Survey, Controlling for Patient Characteristics, Among Veterans With Homeless Experience in HPACT and Mainstream-PACT Primary Care Settings (Showing β-coefficients and Standard Error, SE).

Variable	Model	Model	Model	Model
	PCQ-host relationship (n = 4458)	PCQ-HoST cooperation (n = 4187)	PCQ-HoST access/coordination (n = 4453)	PCQ-HoST homeless-specific needs (n = 4254)
Predisposing				
Race/ethnicity				
White (ref)	—	—	—	—
Latino	0.04 (0.03)	0.04 (0.04)	0.04 (0.03)	0.03 (0.03)
Black	0.04 (0.02)	0.05 (0.03)*	0.04 (0.02)*	0.02 (0.02)
Gender				
Female (ref)	—	—	—	—
Male	−0.03 (0.03)	−0.01 (0.04)	0.01 (0.03)	−0.003 (0.03)
Age				
18-54 (ref)	—	—	—	—
55-65	0.04 (0.02)*	0.10 (0.03)***	0.05 (0.02)**	0.03 (0.02)
66+	0.10 (0.03)***	0.13 (0.03)***	0.09 (0.02)***	0.08 (0.03)**
Enabling				
Medical home				
PACT (ref)	—	—	—	—
HPACT	0.12 (0.02)***	0.15 (0.02)***	0.12 (0.02)***	0.18 (0.02)***
Unsheltered				
No (ref)	—	—	—	—
Yes	−0.09 (0.02)***	−0.10 (0.03)**	−0.08 (0.02)***	−0.17 (0.03)***
Need				
Psychological distress				
No (ref)	—	—	—	—
Yes	−0.15 (0.02)***	−0.24 (0.03)***	−0.16 (0.02)***	−0.17 (0.02)***
Severe chronic pain				
No (ref)	—	—	—	—
Yes	−0.11 (0.02)***	−0.17 (0.02)***	−0.09 (0.02)***	−0.11 (0.02)***
Overdose				
No (ref)	—	—	—	—
Yes	−0.02 (0.03)	−0.02 (0.05)	0.001 (0.03)	0.03 (0.04)

Abbreviations: HPACT: homeless patient aligned care team; PCQ-H: primary care quality-homeless.

*Psychological distress*: Based on combining the 4-item PHQ-4 and 2 items related to psychotic symptoms from the Colorado Mental Health Index, with a score of >10 (sum range 0-24), counted as “Severe” when sum >10, see Methods Section. *Severe Chronic Pain*: Participants who reported having bodily pain that of more than 3 months duration coupled with current pain ≥7 on a 0-10 scale.

*** *P* < .001. **P < .01. *P <. 05. Four models are shown, 1 for each subscale of the Primary Care Quality-Homeless (PCQ-H) survey.

For Cooperation, Black patients had a score that was 0.05-points higher than White patients (1-4 scale). For Access/Coordination, Black patients had a score that was 0.04-points higher than White patients. Scores for Latino patients were nonsignificant (Table 2).

The interaction between clinic type and race/ethnicity was significant only for Cooperation scores. Models (Table 3) suggest being in an HPACT was associated with a greater benefit for White HEVs’ Cooperation score compared to Black HEVs’. In these stratified models, for Black HEVs, HPACT care was associated with a Cooperation score that was 0.09-points higher (standard error (SE) = 0.03) compared to mainstream-PACT. By comparison, White HEVs in HPACT had a Cooperation score 0.19-points higher (SE = 0.04) than their mainstream-PACT counterparts.

**Table 3.** Multivariable-adjusted Ratings of Cooperation on the Primary Care Quality Survey Among Homeless Experienced Veterans in HPACT and Mainstream-PACT Primary Care Settings, Stratified by Race/Ethnicity Group (Each Model Presents β-coefficients and Standard Error, SE, for A Multivariable Model for the PCQ-H Cooperation Score).

Variable	Model (White, n = 1877)	Model (Black, n = 1916)	Model (Latino, n = 565)
Predisposing			
Gender			
Female	—	—	—
Male	0.01 (0.06)	−0.05 (0.06)	−0.08 (0.11)
Age			
18-54 (ref)	—	—	—
55-65	0.16 (0.04)***	0.03 (0.04)	0.19 (0.07)**
66+	0.17 (0.05)**	0.07 (0.05)	0.03 (0.09)
Enabling			
Clinic type			
PACT (ref)	—	—	—
HPACT	0.19 (0.04)***	0.09 (0.03)**	0.08 (0.06)
Unsheltered			
No (ref)	—	—	—
Yes	−0.09 (0.05)	−0.12 (0.05)*	−0.15 (0.09)
Need			
Psychological distress			
No (ref)	—	—	—
Yes	−0.31 (0.04)***	−0.20 (0.04)***	−0.16 (0.07)*
Severe chronic pain			
No (ref)	—	—	—
Yes	−0.22 (0.04)***	−0.13 (0.04)***	−0.20 (0.07)**
Overdose			
No (ref)	—	—	—
Yes	0.04 (0.06)	−0.09 (0.08)	−0.06 (0.11)

Each column represents a new and separate multivariate model.

*Psychological distress*: Based on combining the 4-item PHQ-4 and 2 items related to psychotic symptoms from the Colorado Mental Health Index, with a score of >10 (sum range 0-24), counted as “Severe” when sum >10, see Methods Section.

*Severe Chronic Pain*: Participants who reported having bodily pain that of more than 3 months duration coupled with current pain ≥7 on a 0-10 scale.

*** *P* < .001. **P < .01. *P < .05.

Predisposing, enabling, and need covariates were associated with patient experience across the 4 domains. Being younger and having recent unsheltered experience, severe chronic pain, and psychological distress were strongly and negatively associated with all patient experience ratings (Table 2). Across racial/ethnic groups, at varying effect sizes, severe chronic pain and psychological distress were significantly associated with lower ratings of Cooperation among the primary care team (Table 3).

### Health Service Utilization: Racial/Ethnic Differences in Outpatient

Over a 2-year period, HEVs had an average of 11—primary care, 17—mental health, and 8—SUD clinic visits. The patterns of outpatient utilization were similar across racial/ethnic groups (Table 1). Contrary to expectations, HPACT enrollment was not associated with greater primary care, mental health, or SUD clinic utilization (*P*’s > .05). Moreover, none of the interactions between race/ethnicity and HPACT versus PACT reached statistical significance (*P*’s > .05; interaction results not tabled) for health services utilization.

### Health Service Utilization: Findings Unrelated to Race/Ethnicity

Predisposing, enabling, and need variables were associated with healthcare utilization with unique patterns for each measure (Table 4).

**Table 4.** Multivariable-adjusted 24-month VA Health Service Utilization (Visit Counts) Among Homeless Experienced Veterans in HPACT and Mainstream-PACT Primary Care Settings, With and Without Statistical Adjustment for Patient Experience Measures (Showing β-coefficient and Standard Error, SE).

Health service utilization type modeled						
Variable	Primary care (n = 4,485)	Primary care (n = 4,027) includes statistical adjustment for patient ratings of care	Mental health outpatient (n = 3,438)	Mental health outpatient (n = 3,124) includes statistical adjustment for patient ratings of care	SUD outpatient (n = 2,771)	SUD outpatient (n = 2,525) includes statistical adjustment for patient ratings of care
Predisposing						
Race/ethnicity						
White (ref)	—	—	—	—	—	—
Latino	−0.40 (0.52)	−0.20 (0.53)	0.88 (1.74)	1.51 (1.84)	0.99 (1.64)	1.03 (1.75)
Black	−0.50 (0.36)	−0.44 (0.37)	−1.23 (1.22)	−0.72 (1.28)	0.96 (1.12)	1.33 (1.19)
Gender						
Female (ref)	—	—	—	—	—	—
Male	−2.95 (0.54)***	−2.90 (0.57)***	−1.28 (1.78)	−1.74 (1.91)	1.48 (2.04)	1.37 (2.23)
Age						
18-54 (ref)	—	—	—	—	—	—
55-65	2.46 (0.37)***	2.41 (0.39)***	−0.61 (1.26)	−0.64 (1.33)	0.81 (1.16)	0.87 (1.25)
66+	5.42 (0.46)***	5.01 (0.48)***	−1.76 (1.60)	−1.50 (1.72)	−0.39 (1.55)	−0.49 (1.68)
Enabling						
Clinic type						
PACT (ref)	—	—	—	—	—	—
HPACT	−0.30 (0.32)	−0.19 (0.33)	1.13 (1.11)	0.87 (1.19)	1.26 (1.03)	0.83 (1.12)
Unsheltered (past 6 months)						
No (ref)	—	—	—	—	—	—
Yes	0.05 (0.44)	0.01 (0.45)	3.73 (1.50)*	3.44 (1.58)*	0.35 (1.33)	0.93 (1.42)
Need						
Psychological distress						
No (ref)	—	—	—	—	—	—
Yes	0.43 (0.35)	0.45 (0.26)	3.59 (1.15)**	3.99 (1.22)**	0.35 (1.08)	0.81 (1.16)
Severe chronic pain						
No (ref)	—	—	—	—	—	—
Yes	1.80 (0.33)***	1.75 (0.34)***	−0.66 (1.14)	−0.67 (1.20)	0.11 (1.05)	−0.34 (1.12)
Overdose (past 3 years)						
No (ref)	—	—	—	—	—	—
Yes	1.00 (0.61)	0.65 (0.62)	12.38 (1.99)***	10.55 (2.06)***	10.20 (1.64)***	9.82 (1.73)***
Patient experience ratings						
Relationship		0.46 (0.49)		−2.04 (1.71)		−1.92 (1.63)
Cooperation		−0.26 (0.30)		0.46 (1.06)		0.59 (1.01)
Access/coordination		1.12 (0.56)*		4.37 (1.97)*		1.16 (1.85)
Homeless-specific Needs		−0.16 (0.38)		0.61 (1.35)		1.18 (1.29)

Abbreviation: PCQ-H, Primary Care Quality-Homeless Survey.

Each of the 6 columns presents a multivariable linear model in which the 24-month visit count for the cited type of service is the dependent variable. Results are presented as β-coefficient with Standard Error (SE) in parentheses.

For each of the 3 service types, the model is first iterated without controlling for patient ratings of their care experience, and then with inclusion of those ratings as a covariate.

Mental health outpatient models consist of all persons who had either (a) high psychological distress OR (b) have a mental health diagnosis (look-back period 24 months prior to survey completion date).

Substance use disorder outpatient models consist of persons who had either (a) survey-confirmed SUD on the 2-Item survey screen OR (b) have a SUD diagnoses (look-back period 24 months prior to survey completion).

Models are shown with and without adjustment for patient experience measures:

(a) “Relationship” which captures respondent’s perception of patient-clinician relationship;

(b) “Cooperation” which captures respondent’s perception of cooperation across clinical team;

(c) Access/Coordination which captures respondent’s perception of service accessibility and care team’s coordination; and.

(d) Homeless-specific needs which captures respondents’ perception of their medical home being able to meet their social needs.

Regression analysis indicated that only Access/Coordination was significantly associated (*P* < .05) with outcomes, and was positively associated with both primary care and mental health outpatient visits.

Under “Clinic type,” PACT refers to mainstream primary care, that is, Patient-Aligned Care Teams. HPACT refers to homeless-tailored Patient-Aligned Care teams.

*Psychological distress*: Based on combining the 4-item PHQ-4 and 2 items related to psychotic symptoms from the Colorado Mental Health Index, with a score of >10 (sum range 0-24), counted as “Severe” when sum >10, see Methods Section.

*Severe Chronic Pain*: Participants who reported having bodily pain that of more than 3 months duration coupled with current pain ≥7 on a 0-10 scale.

9. Patient experience ratings are derived from the 4 subscales of the Primary Care Quality-Homeless (PCQ-H) survey.

*** *P* < .001. **P < .01. *P < .05.

#### Primary Care Visits

Male HEVs obtained roughly 3 fewer primary care visits than females. HEVs who were older or reported chronic, severe pain obtained more visits, than HEVs who were younger and reported less pain. When patient experience was added to the model, each 1-point increase in the Access/Coordination rating was associated with 1.12 more primary care visits.

#### Mental Health Visits

Three need variables were associated with having more mental health visits (Table 4): experiencing an overdose (+12.38), being unsheltered (+3.73), and having high psychological distress (+3.59). When patient experience was added, these factors remained significant. These analyses also indicated that greater perceived Access/Coordination was associated with 4.37 additional mental health visits per 1-point increase in score.

#### SUD Clinic Visits

The 1 variable associated with SUD visits was history of overdose (*P* < .05). HEVs reporting an overdose had 10.20 more SUD visits than those who did not. No patient experience ratings were associated with SUD visits.

## Discussion

This study examined primary care experience and outpatient utilization among Black, Latino, and White HEVs and whether HPACT mitigated potential disparities. Few significant differences were found for race/ethnicity by patient experience measures. Across racial/ethnic groups, measures of vulnerability were negatively associated with patient experience. Race/ethnicity was not significantly associated with service utilization, but adding patient experience showed higher Access/Coordination ratings correlated with more primary care and mental health visits.

Black patients reported higher Cooperation and Access/Coordination ratings than White patients, contrasting with prior studies on non-HEVs that found racial disparities linked to between-facility differences.^26,27^ In 1 study, most Black-White differences favored Whites patients, with higher rates of negative experiences at facilities with higher proportions of Black patients, indicating a need for targeted facility-level efforts.^26^ Our study did not focus on facility-level factors, but future research should explore both between- and within-facility influences on HEVs’ experiences. A significant interaction emerged between race/ethnicity and PACT assignment (HPACT vs mainstream-PACT) for Cooperation scores, with score increment twice as large for White than Black patients. While minoritized populations experience disproportionate rates of homelessness, White individuals often cite substance use and psychiatric illness as pathways to homelessness, whereas minoritized groups report structural and economic causes.^28,29^ HPACTs’ focused teams and smaller caseloads may benefit White patients on measures of perceived disagreement or fail to optimize care for Black patients, who have historically faced medical mistreatment and mistrust.^30,31^ Our data alone cannot confirm these inferences, warranting future research.

Regardless of race/ethnicity, higher perceived Access/Coordination was linked to more primary care (+1.12) and mental health outpatient (+4.37) visits, aligning with research on care coordination and engagement.^32,33^ The greater impact of perceived Access/Coordination may be an indication that linkage processes for mental health are critical for patients experiencing homelessness. These findings highlight the benefits of an integrated healthcare system with seamless provider coordination. Additionally, comparisons across race/ethnicity and type of PACT enrollment suggest that HPACT is missing highly vulnerable Latino patients; Latino patients report high psychological distress across PACTs and the highest unsheltered rates in mainstream-PACTs. The reasons for low HPACT enrollment among vulnerable Latino HEVs remain unclear, warranting further research on their clinical and social needs.

This study has limitations. We were unable to capture important SDoH (eg, income and social integration) that may impact primary care experience and healthcare engagement. Our measure of utilization was assessed concurrently with patient experience, limiting causal inferences. Due to small sample sizes, we did not examine other racial/ethnic groups (eg, Asian and Native Americans). Some HEVs may have been missed, as the parent study identified respondents using ICD codes for homelessness.^34^ We also excluded specific diagnoses, though conditions like cardiovascular disease and serious mental illness may drive elevated care engagement. Lastly, we did not analyze facility-level factors (eg, staff diversity and location) that could influence outcomes.

## Conclusions

This is the first study to examine racial/ethnic differences in primary care experience and engagement among HEVs and whether HPACTs offer benefits over mainstream-PACTs for minoritized HEVs. Findings show no significant differences in healthcare experiences or utilization by race/ethnicity; instead, medical and social vulnerabilities were stronger predictors of primary care experiences and service use among HEVs served in VA. Across groups, HPACT was linked to better patient experiences, highlighting the value of tailored care for SDoH (ie, homelessness). HPACT’s success offers insights for non-VA healthcare systems serving vulnerable populations. Additionally, better perceived Access/Coordination correlated with increased primary care and outpatient mental health visits, suggesting benefits to optimization. Findings imply no major racial/ethnic disparities in patient experience or service use among HEVs, laying the groundwork for improving care and monitoring health equity.

## Supplemental Material

sj-docx-1-jpc-10.1177_21501319251382520 – Supplemental material for Comparison of Primary Care Experiences and Outpatient Health Service Utilization Among Black and Latino Homeless-Experienced Veterans: An Analysis of Patient-Centered Medical Homes Supplemental material, sj-docx-1-jpc-10.1177_21501319251382520 for Comparison of Primary Care Experiences and Outpatient Health Service Utilization Among Black and Latino Homeless-Experienced Veterans: An Analysis of Patient-Centered Medical Homes by Melissa Chinchilla, Audrey L. Jones, Aerin DeRussy, Michael F. Green, Lillian Gelberg, Alexander S. Young, Jack Tsai, Sonya E. Gabrielian and Stefan G. Kertesz in Journal of Primary Care & Community Health
