# Supplementary material for: Comparison of Primary Care Experiences and Outpatient Health Service Utilization Among Black and Latino Homeless-Experienced Veterans: An Analysis of Patient-Centered Medical Homes
Source: J Prim Care Community Health. 2025 Nov 9;16:21501319251382520. doi: 10.1177/21501319251382520 (PMC12602919; doi:10.1177/21501319251382520)
Supplement: sj-docx-1-jpc-10.1177_21501319251382520 – Supplemental material for Comparison of Primary Care Experiences and Outpatient Health Service Utilization Among Black and Latino Homeless-Experienced Veterans: An Analysis of Patient-Centered Medical Homes [file sj-docx-1-jpc-10.1177_21501319251382520.docx]

| **Supplemental Table 1.** **Primary Care Quality-Homeless (PCQ-H) patient experience measures** | | | |
| --- | --- | --- | --- |
| **Relationship** | **Cooperation** | **Accessibility / Coordination** | **Homeless-specific Needs** |
| My primary care provider never doubts my health needs. | My primary care and other health care providers need to communicate with each other more. | My primary care provider helps to reduce the hassles when I am referred to other services. | This place tries to help me with things I might need right away, like food, shelter or clothing. |
| My primary care provider takes my health concerns seriously. | I have been frustrated by lack of communication among my primary care and other health care providers. | I have to wait too long to get the health care services my primary care provider thinks I need. | The people who work at this place seem to like working with people who have been homeless. |
| My primary care provider makes decisions based on what will truly help me. | My primary care and other health care providers are working together to come up with a plan to meet my needs. | At this place, I have sometimes not gotten care because I cannot pay. | If I miss an appointment, this place still finds a way to help me. |
| I feel my primary care provider has spent enough time trying to get to know me. |  | If I could not get to this place, I think the staff would reach out to try to help me get care. | At this place, I always have to choose between health care and dealing with other challenges in my life. |
| I can get in touch with my primary care provider when I need to. |  | If I walk-in to this place without an appointment, I have to wait too long for care. |  |
| I can get enough of my primary care provider’s time if I need it. |  | This place is open at times of the day that are convenient for me. |  |
| If my primary care provider and I were to disagree about something related to my care, we could work it out. |  | This place helps me get care without missing meals or a place to sleep. |  |
| My primary care provider makes sure health care decisions fit with the other challenges in my life. |  | It is often difficult to get health care at this place. |  |
| I worry about whether my primary care provider has the right skills to take good care of me. |  | This place tells me about what services are available. |  |
| I can be honest with my primary care provider if I use drugs or alcohol. |  | The health care services I need are close to each other. |  |
| I worry my primary care provider might report my health information to the authorities. |  | If my primary care provider is unavailable there is someone else that can help me. |  |
| Someone from my primary care provider’s office returns my phone calls or pages. |  |  |  |
| When I need information about my health care, like test results, I can get it easily. |  |  |  |
| The staff at this place listens to me. |  |  |  |
| Staff at this place treat some patients worse if they think that they have addiction issues. |  |  |  |
| *Scales are scored using a Likert scale, “strongly agree=4,” “agree=3,” “disagree=2,” “strongly disagree=1,” and “I don’t know =0.” | | | |
